# Effect of irrigation canal conveyance efficiency enhancement on crop productivity under climate change in Nepal

**DOI:** 10.1007/s10661-024-13405-4

**Published:** 2024-11-30

**Authors:** Amit Kumar Basukala, Annette Eschenbach, Livia Rasche

**Affiliations:** 1 Research Unit Sustainability and Climate Risks, Center for Earth System Research and Sustainability (CEN), Universität Hamburg, Grindelberg 5, 20144 Hamburg, Germany; 2 Institute of Soil Science, Center for EarthSystem Research and Sustainability (CEN), University of Hamburg, Hamburg, Germany; 3 Land Use Economics, Universität Hohenheim, Wollgrasweg 43, 70599 Stuttgart, Germany

**Keywords:** Crop modelling, Climate change adaptation, Irrigation water management, Irrigation efficiency, Canal conveyance efficiency

## Abstract

Nepal is expanding its irrigation facilities as an adaptive measure to climate change; however, the current canal conveyance efficiency (CCE) is low with significant water losses. In this study, we assess the potential impact of increasing CCE on the productivity of rice, maize, and wheat under different climate change scenarios (SSP1-2.6, SSP3-7.0, and SSP5-8.5), utilizing three bias-adjusted general circulation models. The study simulates potential yields at ecoregion levels for two periods: near future (2023 to 2050) and end-century (2075 to 2100). Management scenarios include the following: (1) business as usual, (2) CCE at 30%, (3) CCE at 50%, and (4) CCE at 70%. The results indicate that increasing CCE to 30%, coupled with expanded irrigated areas and adjusted fertilization rates, could boost yields by three tons per hectare across all three crops at the national level. Further increasing CCE to 50% could yield additional increases of up to 0.6 t/ha of maize and 1.2 t/ha of rice in the terai region. A CCE of 70% results in further increases of up to 2.1 t/ha of rice and 1.2 t/ha of maize. The benefits of improved CCE vary by location, with the subtropical terai region experiencing the most and the mountain regions showing the least. We conclude that there is potential to increase yields by increasing CCE to 70% in the terai region, 50% in the hill region, and 30% in the mountains. Wheat appears to benefit the least from improved CCE. This work highlights efficient irrigation as a reliable adaptive measure for future climate change in Nepal.

## Introduction

Worldwide food production must be doubled by 2050 to fulfil the prerequisites of the growing human population (Rosa et al., 2020). Irrigated agriculture, which accounts for 40% of global food production, occupies only 22% of the total cultivated land (Beltran-Peña et al., 2020). Projections suggest that the extent of global irrigated agriculture will need to double by 2050, which will significantly increase the demand for irrigation water (Puy et al., 2020). Currently, half of the water used for irrigation is unsustainable, surpassing the local renewable water supplies and negatively impacting water flows both locally and downstream in various regions (Rosa et al., 2019). This decreased water availability results in water insecurity, especially in the context of climate change (Kaini et al., 2021), which has direct impacts on spatial and temporal patterns in precipitation and temperature (Kaini et al., 2020a, 2020b). Hence, there is a need for the implementation of efficient and sustainable irrigation systems to ensure the long-term sustainability of agriculture and the health of the environment.

Adopting sustainable irrigation practices in a world facing water scarcity involves reducing water losses in irrigation systems, decreasing the demand for irrigation water, and improving water productivity. In irrigation systems, significant conveyance water losses are caused by seepage through canal bunds, deep percolation in the canal and field soil layers, runoff into the drain, and evaporation from the water surface (Eltarabily et al., 2020). Factors such as soil permeability, the depth of water channels, the length of the wetted perimeter, water table level, and flow velocity contribute to these conveyance losses (Abd-Elaty et al., 2022; Nam et al., 2016). Canal lining with materials like concrete, geomembrane, bentonite, polyvinyl chloride, or plastic can significantly enhance canal conveyance efficiency by minimizing water loss before it reaches the fields (Han et al., 2020). This strategy not only increases the availability of water for irrigation but also improves water quality (Nofal et al., 2024). Water conservation through canal lining ensures equitable and reliable water distribution to farmers, leading to higher crop yields and cropping intensity. It also helps in controlling waterlogging, preventing erosion and breaches along channel banks, and reducing operational and maintenance costs (Singh, 2017).

**Fig. 1:**
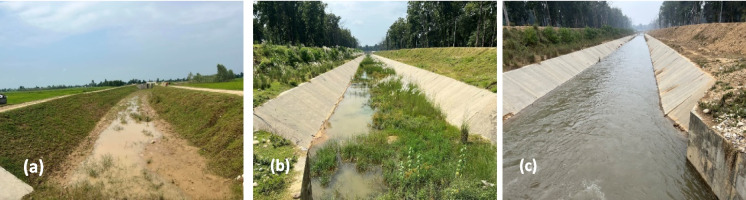
**a** Earthen canal with high seepage and percolation loss (low conveyance efficiency). **b** Concrete lined canal but not well maintained (low conveyance efficiency). **c** Well-maintained canal lined with concrete (high conveyance efficiency) of Lamki Extension Canal of Rani Jamara Kulariya Irrigation Project

Gravity water flow through long canal systems has been a primary method for transporting water for irrigation in Nepal. These canals are notably inefficient in terms of canal conveyance efficiency (Fig. 1a and 1b) (DoWRI, 2019). Most of the canals are earthen structures (Fig. 1a), resulting in significant water loss during conveyance from the canal head to the farmers’ fields (ADB-Mid-Hill-Project, 2016; Kaini et al., 2024). Addressing seepage and percolation losses in these canals (Fig. 1c) could potentially allow for the irrigation of additional agricultural land. Presently, irrigation efficiency in Nepal is below 30%, and the Department of Water Resources and Irrigation aims to increase this efficiency to 40% by 2045 (DoWRI, 2019). Consequently, the Government of Nepal has not only prioritized expanding the area under irrigation but also aims to achieve an irrigation efficiency of 50% by 2027, as outlined in its National Water Plan-Nepal (WECS, 2005). In the context of Nepal’s underperforming agricultural sector (Basukala & Rasche, 2022), developing an efficient irrigation infrastructure and ensuring an effective operation and maintenance of existing systems is crucial for boosting agricultural production. This is especially important considering the challenges faced in constructing new irrigation systems, such as rising capital costs, adverse topography, inadequate infrastructure, and political instability.

There are studies related to the evaluation of efficiency of surface irrigation systems (Kulkarni & Nagarajan, 2019; Lamichhane et al., 2022; Shumye & Singh, 2018), calculation canal conveyance losses (Liao et al., 2022; Luo et al., 2015; Syed et al., 2021), and minimizing these losses (Barkhordari et al., 2020; Cui et al., 2023; Elkamhawy et al., 2021; El-Molla & El-Molla, 2021; Josiah et al., 2016; Kalybekova et al., 2023; Lamichhane et al., 2022; Yuguda et al., 2020). Additional research has focused on failure mechanisms and treatment measures for canals (Chen et al., 2023), and the economic aspects of irrigation efficiency in relation to fuel consumption (Baral et al., 2022). However, we are not aware of any studies that assess the impact of varied canal conveyance efficiencies on crop yields, particularly through biophysical crop models that consider the perspective of climate change.

Similarly, there are studies exploring the expansion of irrigation related to crop production and irrigation water demand under various climate scenarios for crops like maize (Liao et al., 2024; Shan et al., 2023; Yan & Du, 2023; Yetik & Sen, 2023), wheat (Acharjee & Mojid, 2023; Gao et al., 2024; Haymale et al., 2020; Kaini et al., 2022; Luo et al., 2022; Peng et al., 2019; Rowshon et al., 2019; N. Wang et al., 2022a, 2022b; Yan & Du, 2023), and rice (Houma et al., 2021; Kulyakwave et al., 2023; Sun et al., 2024). However, the effects of different canal conveyance efficiencies on crop yield are not accounted for in these studies. Therefore, this study aims to investigate the effects of climate change on rice, wheat, and maize crop yields across different Shared Socioeconomic Pathway (SSP) scenarios, taking into account water availability based on varying canal conveyance efficiencies. Our analysis covers timeframes for the near future (2022–2050) and the end of the century (2075–2100) using low (SSP1-2.6), high (SSP3-7.0), and extreme (SSP5-8.5) emissions scenarios. We utilize three general circulation models (GFDL-ESM4, IPSL-CM6A-LR, and MPI-ESM1–2-HR) sourced from the ISIMIP3b database and bias-adjusted as part of CMIP6. This data, derived from ensembles of global circulation models, represents various climatic conditions. Our research aims to address the following research questions:

i. How will expanding irrigation as a result of increasing canal conveyance efficiency (CCE) to 30% influence future crop yields?
ii. How will improving CCE from 30 to 50% affect crop yields under current to near-future climate conditions?
iii. How will an increase in CCE from 30 to 50% impact crop yields under climate conditions projected for the end of the century?
iv. What are the projected benefits in terms of crop yields when increasing CCE to 70%?

This study aims to support decision-making related to the expansion of irrigation, focusing on the appropriate conveyance efficiency of canal systems for the major crops rice, maize, and wheat across the diverse ecoregions of Nepal.

## Materials and methods

### Study area

**Fig. 2:**
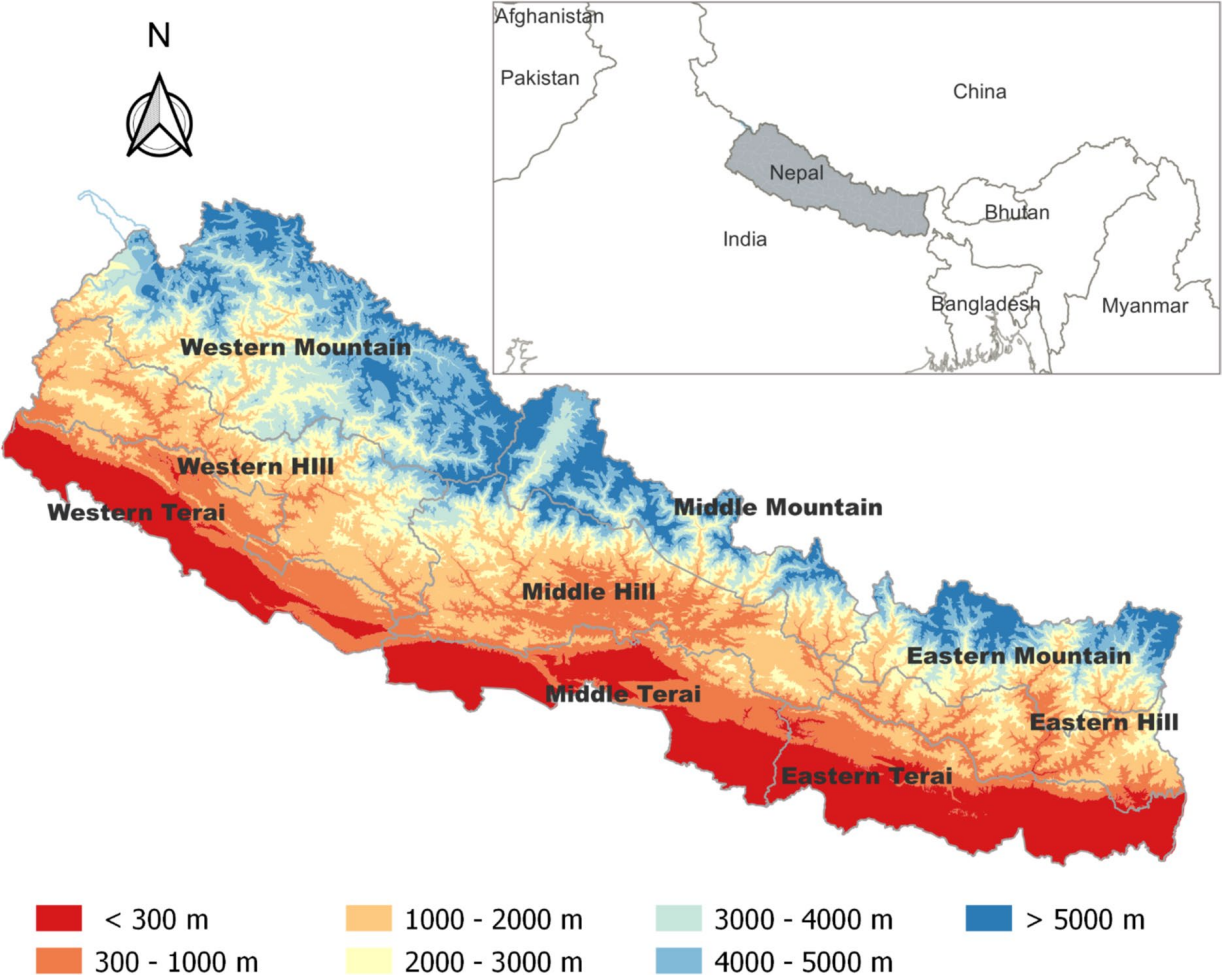
The nine different agro-ecological zones of Nepal with different elevation classes. The enclosure at the top right shows the location of Nepal in South Asia with neighbouring countries

Nepal is a South Asian Himalayan nation situated between China and India with geographical coordinates between 26° 22′ N and 30° 27′ N latitude and between 80° 04′ E and 88° 12′ E longitude, and a spatial coverage of 147,181 km^2^. It has a population of approximately 30.5 million as of 2022 (WorldBank, 2022), and a wide range of ecological regions, including tropical, subtropical, temperate, subalpine, alpine, and nival zones (Paudel et al., 2021). Due to the sharp topographical constrasts (Fig. 2), Nepal has diverse rainfall patterns: mean annual precipitation ranges from under 150 to over 5000 mm. Monsoons contribute roughly 80% of the annual precipitation, with the winter and pre- and post-monsoon seasons contributing 3.5%, 12.5%, and 4.0%, respectively (Karki et al., 2017). The spatial distribution of annual precipitation follows the monsoon pattern (Department of Hydrology and Meteorology (DHM), 2015). The hydrology of the Himalayan region is rapidly changing due to climate change (Talchabhadel & Chhetri, 2023). Nepal’s economy relies heavily on agriculture, which provides livelihoods for about 68% of the population. Unfortunately, the agricultural sector in Nepal has been struggling since 1995, leading to the need for food imports to ensure food security (Bigyan et al., 2021). The reason for the underperforming agriculture sector of Nepal is the unavailability and low application rates of both organic and inorganic fertilizers, prevalent dependency on rainfall for irrigation, poor agricultural infrastructure, land abandonment, and vulnerability to external shocks such as climate change-induced erratic rainfalls, floods, and prolonged droughts (Basukala & Rasche, 2022; Bocchiola et al., 2019; Joshi et al., 2021). The total cultivated land in Nepal covers 41,210 km^2^, primarily concentrated in the flat Southern region known as the ‘terai’. The middle hill region consists of fertile valleys, hilly peaks, and river basins, with only a small fraction of the land suitable for cultivation. The northern mountain region has a mere 2% of cultivable land. Rice, wheat, and maize are the three important cereal crops in Nepal, collectively contributing 31% of the agricultural GDP (Ministry of Agricultural Development (MOAD), 2022). As of 2015, 52% of agricultural land was irrigated, with year-round irrigation available in 36% of the area (Pokhrel, 2019).

### Data

#### Climate data

We utilized daily weather parameters encompassing precipitation (in mm/day), maximum and minimum temperatures (in °C), relative humidity (fraction), solar radiation (in MJ/m^2^), and wind speed (m/s) for three distinct periods: a baseline (2015–2021), a near future (2022–2050), and a far future (2075–2100). The future climate projections incorporate three scenarios derived from phase 6 of the Coupled Model Intercomparison Project (CMIP6). The scenarios in our study encompass low-end (SSP126), medium–high (SSP370), and high-end (SSP585) future forcing pathways. Bias-corrected and statistically downscaled climate data from phase 3b of the Inter-Sectoral Impact Model Intercomparison Project (ISIMIP3b) (Lange, 2019) were used for all scenarios. We used three CMIP6 models: GFDL-ESM4, MPI-ESM1-2-HR, and IPSL-CM6A-LR. The dataset has a spatial resolution of 0.44°. The ISIMIP3b climate data were downloaded from the ISIMIP repository (https://data.isimip.org/search/).

**Fig. 3:**
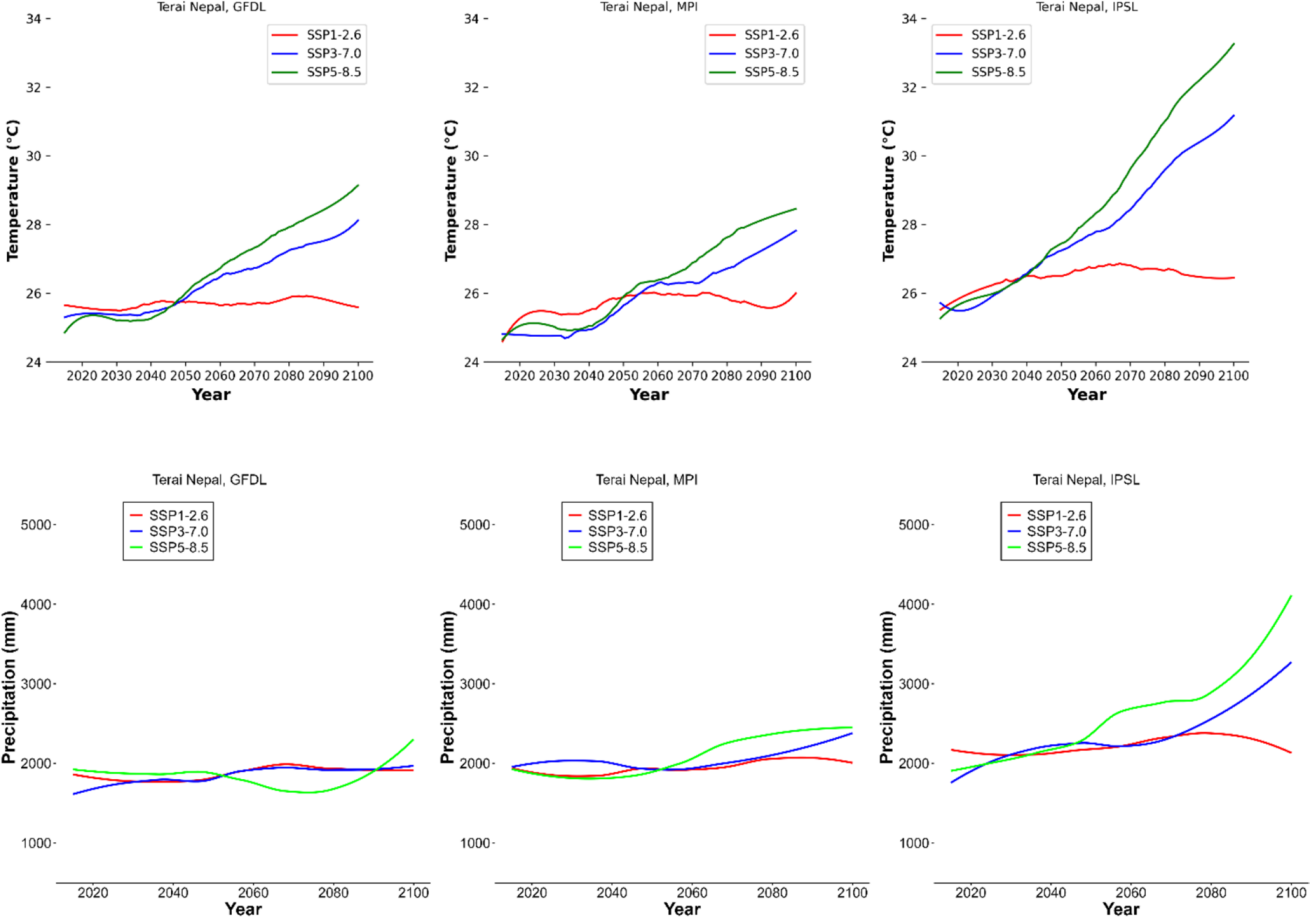
First row: trends of annual average temperatures in the terai ecoregion under three different climate scenarios using three different general circulation models; second row: trends of annual precipitation in the terai ecoregion under three different climate scenarios using three different general circulation models

In the terai region, GFDL predictions for SSP5, SSP3, and SSP1 scenarios show temperature rises of 3.9 °C, 2.8 °C, and a decline of 0.2 °C, respectively. The MPI model anticipates temperature changes of 3.3 °C (SSP5), 2.8 °C (SSP3), and 0.8 °C (SSP1) in the area (Fig. 3). In the hill region, projections from the GFDL climate model for SSP5, SSP3, and SSP1 scenarios suggest temperature increases of 3.8 °C, 3.3 °C, and 0.5 °C, respectively. Correspondingly, the MPI model predicts temperature elevations of 3.6 °C (SSP5), 3.1 °C (SSP3), and a decline of 0.5 °C (SSP1) in the same region. The IPSL model forecasts higher increments, with temperature changes of 7.7 °C (SSP5), 5.1 °C (SSP3), and decline of 0.9 °C (SSP1) at the hill region (Fig. 15). For the Mountain region, GFDL model projections show a temperature rise of 4.2 °C (SSP5), 3.7 °C (SSP3), and a decrease of 0.3 °C (SSP1). The MPI model indicates temperature changes of 4.3 °C (SSP5), 3.2 °C (SSP3), and 0.8 °C (SSP1). The IPSL model predicts increases of 8.5 °C (SSP5), 6.3 °C (SSP3), and 0.4 °C (SSP1) within this region (Fig. 16).

By the end of the century, based on the GFDL climate model, the annual precipitation will increase by 130 mm, 440 mm, and 300 mm for SSP1-2.6, SSP3-7.0, and SSP5-8.5, respectively in the terai region (Fig. 3). Under the MPI climate model, the annual precipitation will increase by 190 mm, 282 mm, and 533 mm, and by 214 mm, 1092 mm and 1950 mm under the IPSL climate model for SSP1-2.6, SSP3-7.0, and SSP5-8.5, respectively. In the terai region, precipitation is  279 mm lower in scenario SSP3 compared to scenario SSP1, and 665 mm higher in scenario SSP5 (Fig. 3). Similarly, in the hill region, precipitation under SSP3 is 255 mm lower than under SSP1, and 474 mm higher under SSP5 (Fig. 17), while in the mountain region, precipitation is 342 mm lower under SSP3 than under SSP1, and 705 mm higher under SSP5 (Fig. 18).

#### Soil, elevation, and slope data

The soil types data for each simulation unit were derived from the global dominant soil typological units (STUs) outlined in the GEOBENE database (Skalský et al., 2008). STUs are derived from the FAO Digital Soil Map of the World (DSMW) and the World Inventory of Soil Emission Potentials (WISE) database. The STUs provide data on the soil characteristics the biophysical model requires, e.g. bulk density, electric conductivity, water content at field capacity and wilting point, pH, and cation exchange capacity (Jones & Thornton, 2015). The elevation and slope data for the study area were procured from a topographic map of the NASA Shuttle Radar Topographic Mission (SRTM) 90m Digital Elevation Database (v4.1). This resource, accessible through the Consortium for Spatial Information (CGIAR-CSI) under the Consultative Group for International Agricultural Research (CGIAR), offers a resolution of 90m at the equator (Jarvis et al., 2008).

#### Management data

Information regarding rice, wheat, and maize crop areas and yields, spanning the fiscal years 1990 to 2022, were sourced from Nepal’s agricultural statistics for cereal crops (Ministry of Agricultural Development (MOAD), 2022). Data regarding fertilizer and irrigation applications per hectare (Table 10), including application timings, were obtained from Nepal’s CBS decadal agriculture census records for 2011, 2021, and Takeshima (2019). For Nepal’s key crops, the district-specific crop calendar details were sourced from an FAO/WFP food security assessment mission to Nepal (Table 11).

### Derivation of simulation units

Simulation units are the smallest distinct areas within a study area where data inputs and outputs are managed independently to simulate specific processes. We simplified the representation of Nepal’s diverse agriculture landscape, spatial heterogeneity, and climatic conditions by creating Homogenous response units (HRUs). HRUs were used to represent areas within a landscape that have uniform characteristics affecting crop growth and yield. We followed the method used for constructing the Global Earth Observation-Benefit Assessment (GEOBENE) database (Skalský et al., 2008). The classification involved sorting the territory into seven elevation classes, seven slope classes, and ten soil classes (Table 12), ensuring a consistent and standardized representation for simulation purposes.

**Fig. 4:**
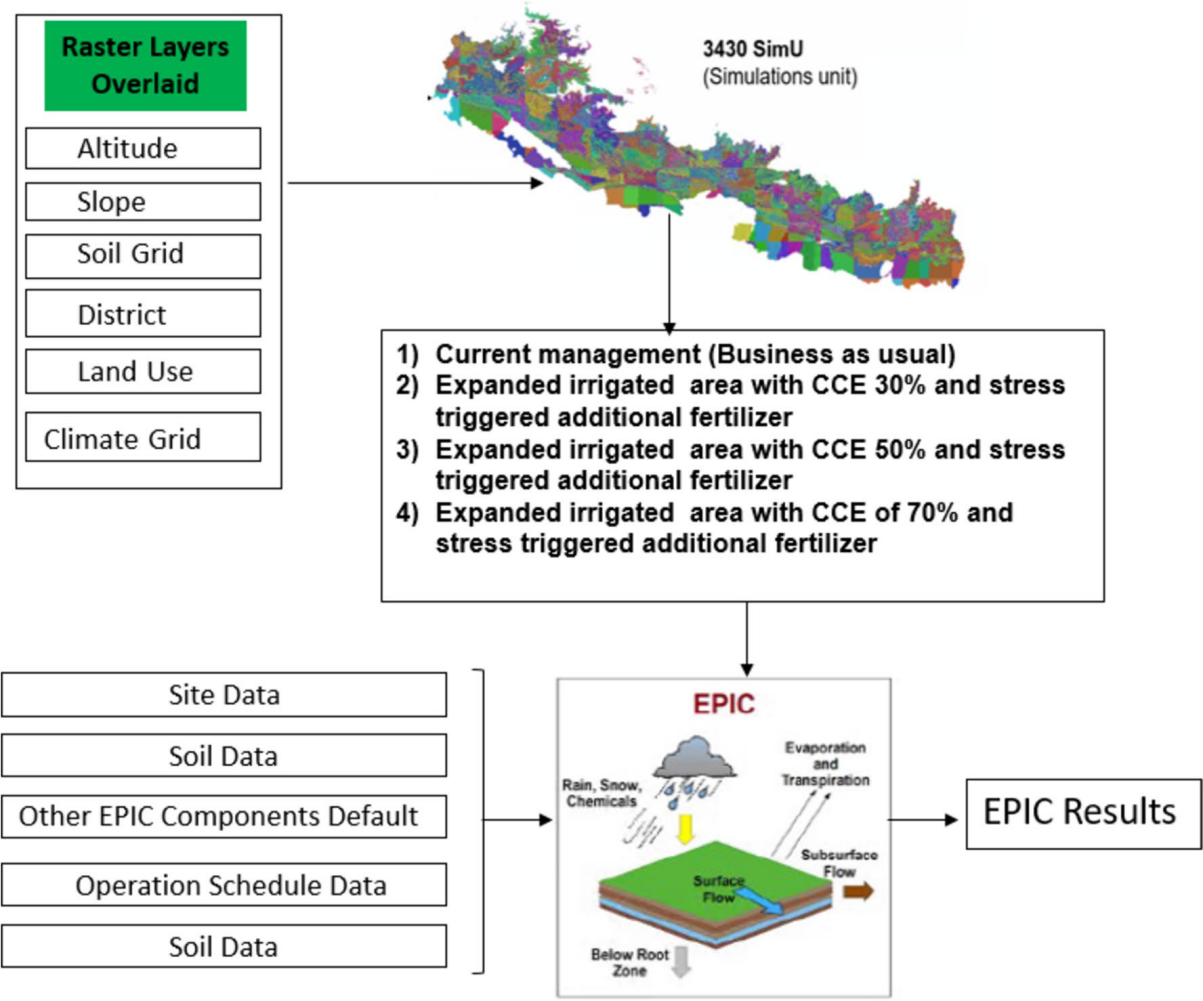
Structural diagram of the methodological framework used for the study of the effect of irrigation canal conveyance efficiency enhancement on crop productivity under climate change in Nepal

After demarcating the HRUs, we proceeded to subdivide these units based on district boundaries, land use, and land cover, as well as the climate data raster. Subsequently, we identified cropland and non-cropland areas through the land use mask and excluded the non-cropland areas from the simulations, thus creating 3430 SimUs (Fig. 4). To discuss the results, we also divided the country into nine sub-regions based on physiography and longitude (cf. Figure 1). To define district boundaries and nine sub-regions, we sourced GIS data from the Global Administrative Areas database (GADM, 2018). The land use mask utilized was extracted from the Land Cover of Nepal 2010 dataset, developed by the International Center for Integrated Mountain Development (ICIMOD) (Uddin et al., 2015).

### Crop model

We utilized the Environmental Policy Integrated Climate (EPIC) model for the crop simulations (Williams et al., 1989). EPIC is a complex biophysical process-based model designed to assess the interactions between agricultural systems, climate dynamics, and environmental conditions. EPIC combines meteorological data, soil information, and crop-specific parameters to simulate various aspects of agricultural processes. Through its comprehensive modeling framework, EPIC can predict crop growth, yield, water usage, nutrient dynamics, soil erosion rates, and greenhouse gas emissions (Di Bene et al., 2022; Gaiser et al., 2010).

Plant growth in EPIC is simulated via intercepted solar radiation and the ability of each plant to convert the intercepted radiation into biomass. The leaf area index (LAI) represents the total area of leaves in relation to the ground area, providing insights into a plant’s ability to capture sunlight for photosynthesis (Zhang et al., 2018). Biomass, the accumulated organic material produced by plants, is a key output of the EPIC model. The potential daily biomass increase may be reduced if the plant is water-, temperature- or nutrient-stressed. The crop yield estimation employs the harvest index principle, where the harvest index rises nonlinearly with accumulated heat units from zero at planting to the optimal value at maturity (Williams et al., 1989). However, high temperatures, low solar radiation, or water stress during critical crop stages can diminish the harvest index (Yang & Zhang, 2010). Additionally, EPIC offers flexibility by providing a selection of five potential evapotranspiration equations (Balkovič et al., 2013), diverse crop rotations, tillage systems, and management practices.

We opted for the utilization of the EPIC model in this study due to its consistently reliable performance across diverse settings (Z. Q. Wang et al., 2022a, 2022b), especially considering the challenging environmental conditions present in Nepal for crop simulations. The outputs generated by the EPIC model offer comprehensive details on crop growth, water dynamics, nutrient interactions, and carbon fluxes at daily, monthly, and yearly intervals. In this investigation, our emphasis is on key parameters such as the estimated annual yield, and the annual irrigation water supply.

### Model calibration

We first calibrated the yields of rice, wheat, and maize for the baseline period (2015–2021). The initial crop parameter values were derived from the standard crop parameter set in the EPIC crop database file. Calibration focused on crop-specific parameters such as potential heat units, radiation use efficiency, harvest index, and optimal and base temperature. We began the calibration by running the model separately for each crop. The resulting simulated crop yields were compared against the reported district-wise yield data for rice, wheat, and maize from the agricultural statistics of cereal crops in Nepal (Ministry of Agricultural Development (MOAD), 2022) for the years 2015–2021. We then repeatedly adjusted the aforementioned crop-specific parameters to minimize the discrepancies. The range of crop parameter values for iterations was restricted, we did not allow it to deviate by more than 20% from the original values or the range defined as feasible in the EPIC user guide. Through this process, we fine-tuned the crop parameters to match the local crop variety characteristics. We could not identify a single set of crop parameters applicable to all regions of Nepal and therefore decided to derive a separate crop parameter set for each of the 77 districts in Nepal.

A total of four statistical measures were used to assess the adequacy of fit of the crop model during calibration: (i) Nash Suttcliffe Effieciency (NSE), (ii) correlation coefficient value (r), (iii) relative error (RE), and (iv) percent bias (PBIAS) as shown in Table 1. The Nash–Sutcliffe efficiency quantifies the agreement between reported and simulated crop yield providing a measure of model accuracy. The percent bias identifies the average affinity of the simulated crop yield to be larger or smaller than the reported yield. The correlation coefficient is an indicator that evaluates the agreement between simulated and reported crop yields. The relative error measures the size of the error in relation to the reported value, providing insight into the accuracy of the model’s predictions.

**Table 1:** Measure of agreement between average simulated crop yields with average reported crop yields

Name	Equation	Optimum value
Nash–Sutcliffe efficiency (NSE)	$NSE=1-\frac{{\sum }_{i=1}^{n}{\left({X}_{i}-{\overline{\text{Y}} }_{i}\right)}^{2}}{{\sum }_{i=1}^{n}{\left({X}_{i}-\overline{\text{X} }\right)}^{2}}$	1
Percent bias (PBIAS)	$PBIAS=1-\frac{{\sum }_{i=1}^{n}\left({Y}_{i}-{\text{X}}_{\text{i}}\right)*100}{{\sum }_{i=1}^{n}{Y}_{i}}$	0%
Correlation coefficient (r)	$r=\frac{{\sum }_{i=1}^{n}\left({X}_{i}-\overline{\text{X} }\right)\left({Y}_{i}-\overline{Y }\right)}{\sqrt{{\sum }_{i=1}^{n}{\left({X}_{i}-\overline{\text{X} }\right)}^{2} {\sum }_{i=1}^{n}{\left({Y}_{i}-\overline{Y }\right)}^{2}}}$	1
Relative error (RE)	${\text{RE}}_{\text{i}}=\frac{\left({\overline{\text{Y}} }_{\text{i}}-{\text{X}}_{\text{i}}\right)}{{\text{X}}_{\text{i}}}\cdot 100$	0%

In the above equations, X_i_ is the reported crop yield in district i, Y_i_ simulated crop yield in district i, and n is the total number of districts, $\overline{\text{X} }$ is the simulated crop yield mean and $\overline{\text{Y} }$ is reported crop yield mean, $\overline{\text{Y} }$_i_ is the simulated average crop yield in district i.

### Calculation of future irrigation requirements

We conducted simulations of irrigation requirements over a 78-year period from 2022 to 2100 for each simulation unit and each crop, drawing inspiration from Wriedt et al. (2009) for their methodology in estimating irrigation needs in Europe. During the simulation, additional irrigation was automatically applied on days when plants experienced a moderate level of water stress (stress factor of 15% or higher). For each district, the net irrigation requirement in mm was determined by calculating the area-weighted average of the net irrigation requirements from all simulation units for the near future and end-of-century periods. The irrigation volumes applied to the fields in each scenario were then determined according to the percentages of water conveyance efficiency of the irrigation systems (30%, 50%, and 70%); mean values for each ecoregion are shown in the supplementary materials, Figs. 12, 13, 14. These calculated irrigation amounts were subsequently integrated into the scenarios run in the EPIC model. Water conveyance efficiency refers to the ratio between the water that reaches a field and that is diverted from the irrigation water source.

1 $${\text{E}}_{\text{c}}=\frac{{\text{V}}_{\text{f}}}{{\text{V}}_{t}}\cdot 100$$

In this equation, E_c_ is the water conveyance efficiency, V_f_ is the volume of water applied to the land, and V_t_ is the volume of water diverted from the source.

### Simulation scenarios

Irrigation and fertilizer applications explain 60–80% of the global yield variability for most major crops (Muller et al., 2019), which is why we consider these two factors explicitly in our scenarios and ignore other factors such as changes in tillage, mulching, pest control, or cultivar development. The four scenarios are as follows:

1. Current management (business as usual)
2. Expanded irrigated area with water conveyance efficiency of 30%, additional fertilizer if required
3. Expanded irrigated area with water conveyance efficiency of 50%, additional fertilizer if required
4. Expanded irrigated area with water conveyance efficiency of 70%, additional fertilizer if required

The expansion of the irrigated area implies that irrigation is allowed on all simulation units if the plant experiences water stress. Under current management, irrigation is only allowed on the simulation units where irrigation is currently practiced. The additional fertilizer applications in scenarios 2, 3, and 4 were triggered automatically during the simulation if the plant experienced a moderate amount of nutrient stress on a specific day (stress factor higher or equal to 15%). For scenarios 2, 3, and 4, stress-triggered mineral phosphorous applications and a maximum annual amount of 300 kg of mineral nitrogen per hectare were allowed. Irrigation was allowed up to the district-wise maximum irrigation calculated for each CCE in Sect. 2.5. Each scenario was run for all 3430 simulations units covering Nepal for the periods 2015 to 2050 and 2075 to 2100.

## Results

### Calibration of crop yields

Crop yields were simulated under reported management practices from 2015 to 2022 across all simulation units. District, province, ecological zone, and national level crop yields were derived by aggregating the yields of all simulation units within their respective spatial boundaries. The simulated mean crop yields at the national level were within the same range as the reported mean crop yields: rice yielded 3.7 t/ha versus the reported 3.4 t/ha, wheat 2.1 t/ha versus the reported 2.5 t/ha, and maize 2.63 t/ha versus the reported 2.68 t/ha. At the district level, simulated rice yields were in some instances slightly higher than reported yields, yet overall they remained comparable. For wheat, simulated yields were lower than reported yields in certain cases but were generally within the same range. Maize yields, both simulated and reported, demonstrate a performance that is notably better compared to the other two crops, and their results align closely within each other. The Nash–Sutcliffe efficiency (NSE) values were 0.54 for wheat, 0.59 for rice, and 0.72 for maize. These values indicate that the simulation model performed more effectively in predicting crop yields compared to a simple average of the observed yields across all crops. The percent bias (PBIAS) between reported and simulated mean annual yields was − 7.2% for rice, 1.1% for maize, and − 12.3% for wheat.

**Fig. 5:**
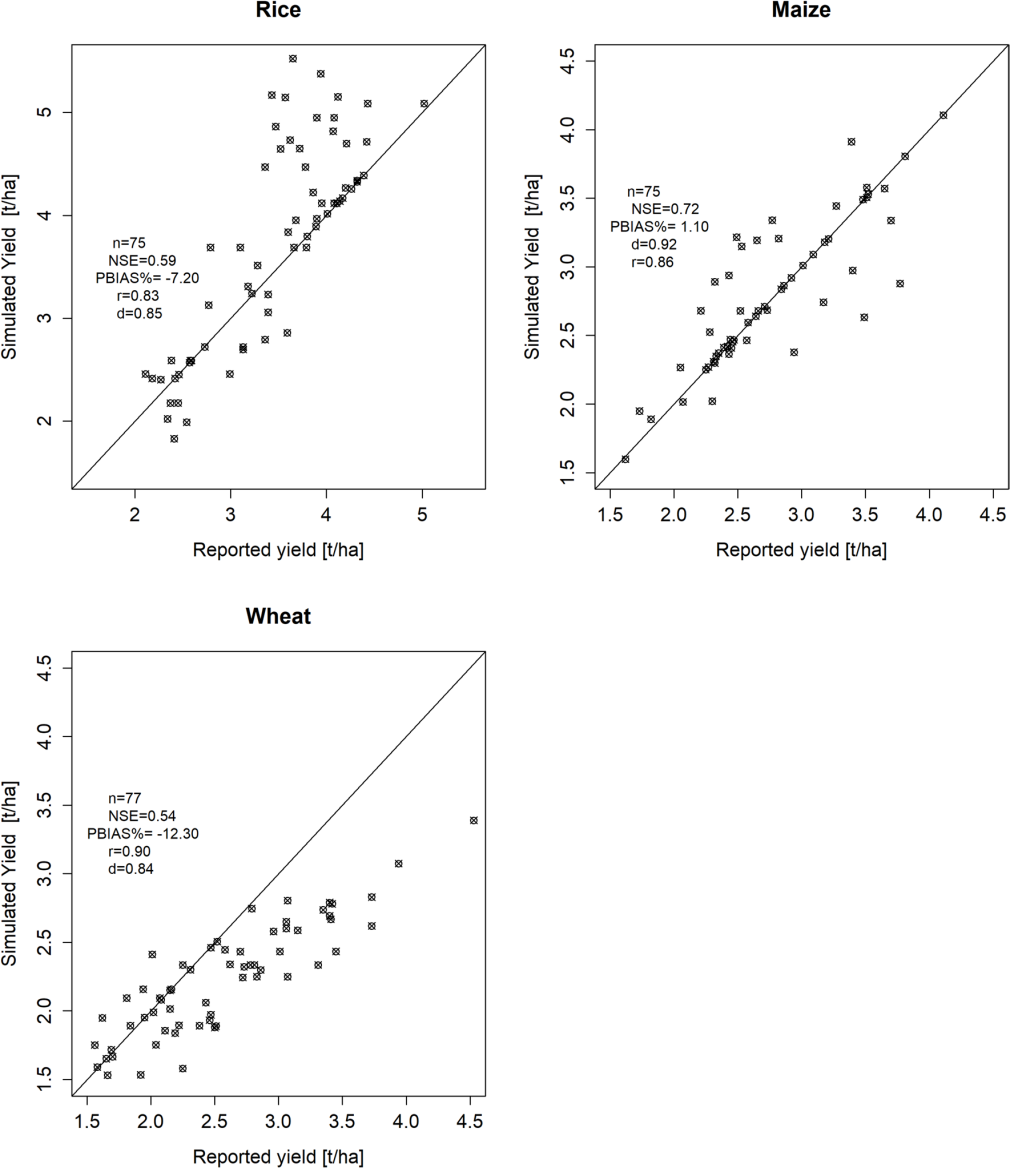
Association of district-level average simulated crop yields with average reported crop yields published by the Ministry of Agriculture Development (MOAD) Nepal. The line indicates perfect agreement where simulated crop yields (x) = average reported crop yields (y). Every single point represents an average yield over the period from 2015 to 2021. NSE signifies Nash–Sutcliffe efficiency; PBIAS signifies percentage bias, n the number of districts, r the Pearson correlation coefficient, and d the degree of agreement

The correlation coefficient *r* was 0.83 for rice, 0.86 for maize, and 0.9 for wheat, which indicate a strong positive linear relationship between reported and simulated crop yields for all three crops. The degree of agreement, as measured by the index of agreement, ranged from 0.84 for wheat and 0.85 for rice to 0.86 for maize, indicating a high level of consistency between the compared data sets. The relative error (RE) values spanned from − 35.02 to 31.07% for rice, − 41.1 to 48.1% for wheat, and − 44.21 to 29.14% for maize. These values represent the measure of accuracy of the numerical approximations as compared to both the reported and simulated values. Based on the statistical indicator values, the calibration can be considered satisfactory. The scatterplot illustrates that our calibrated EPIC model slightly overestimated the average rice yield, underestimated the average winter wheat yield, but provided a good estimate for the maize yield in Nepal (Fig. 5).

### Crop yield development under different scenarios in the different ecoregions of Nepal

To assess the absolute variations in crop yield values across different Shared Socioeconomic Pathway (SSP) scenarios and canal conveyance efficiency (CCE) scenarios, we aggregated the results to the nine ecoregions.

#### Rice

**Fig. 6:**
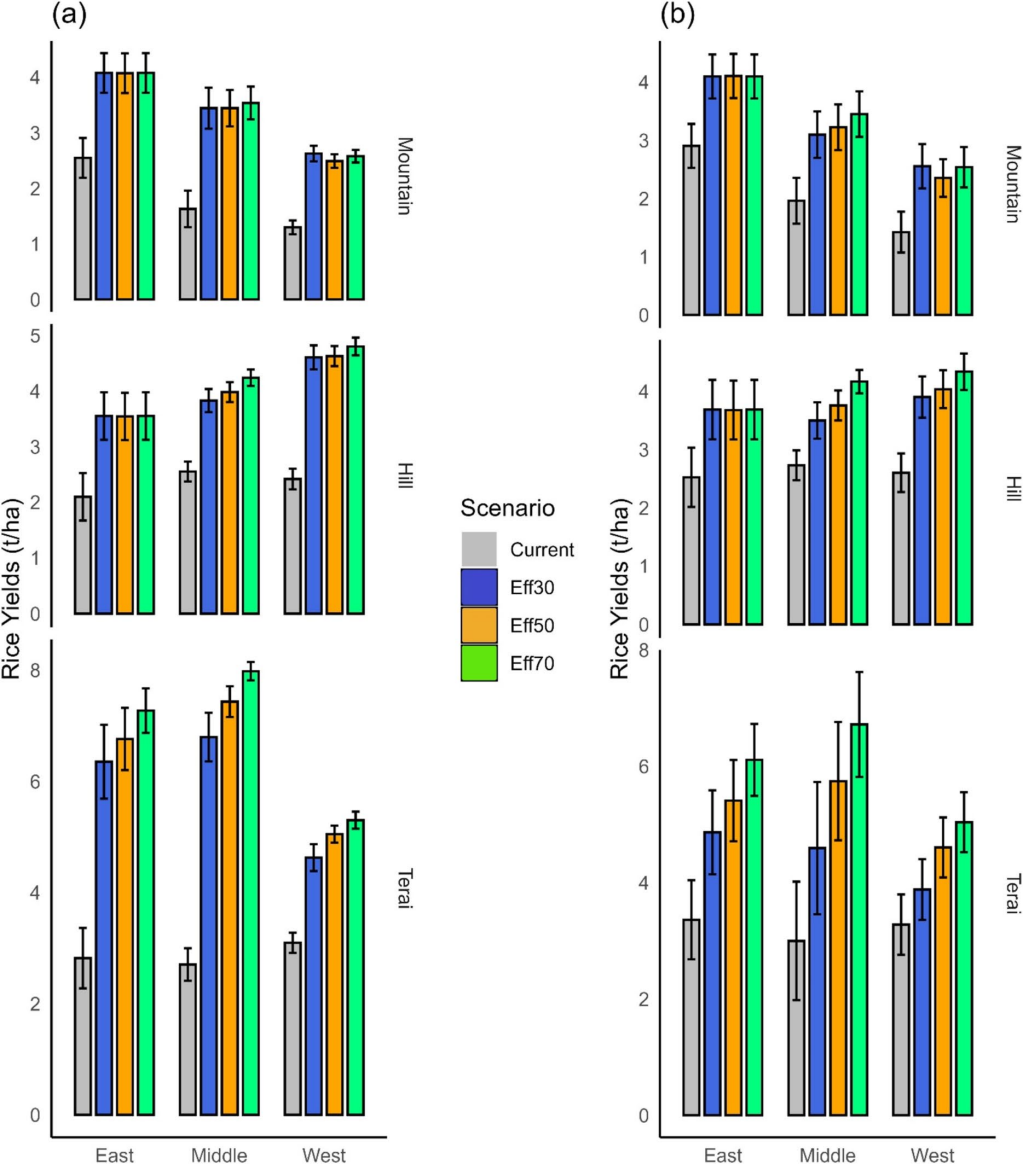
Rice yields in the near future (**a**) and far future (**b**). Eff30 = canal conveyance efficiency (CCE) of 30%, Eff50 = CCE of 50%, Eff70 = CCE of 70%. The confidence intervals denote the range over the three climate change scenarios and the three GCM models

**Fig. 7:**
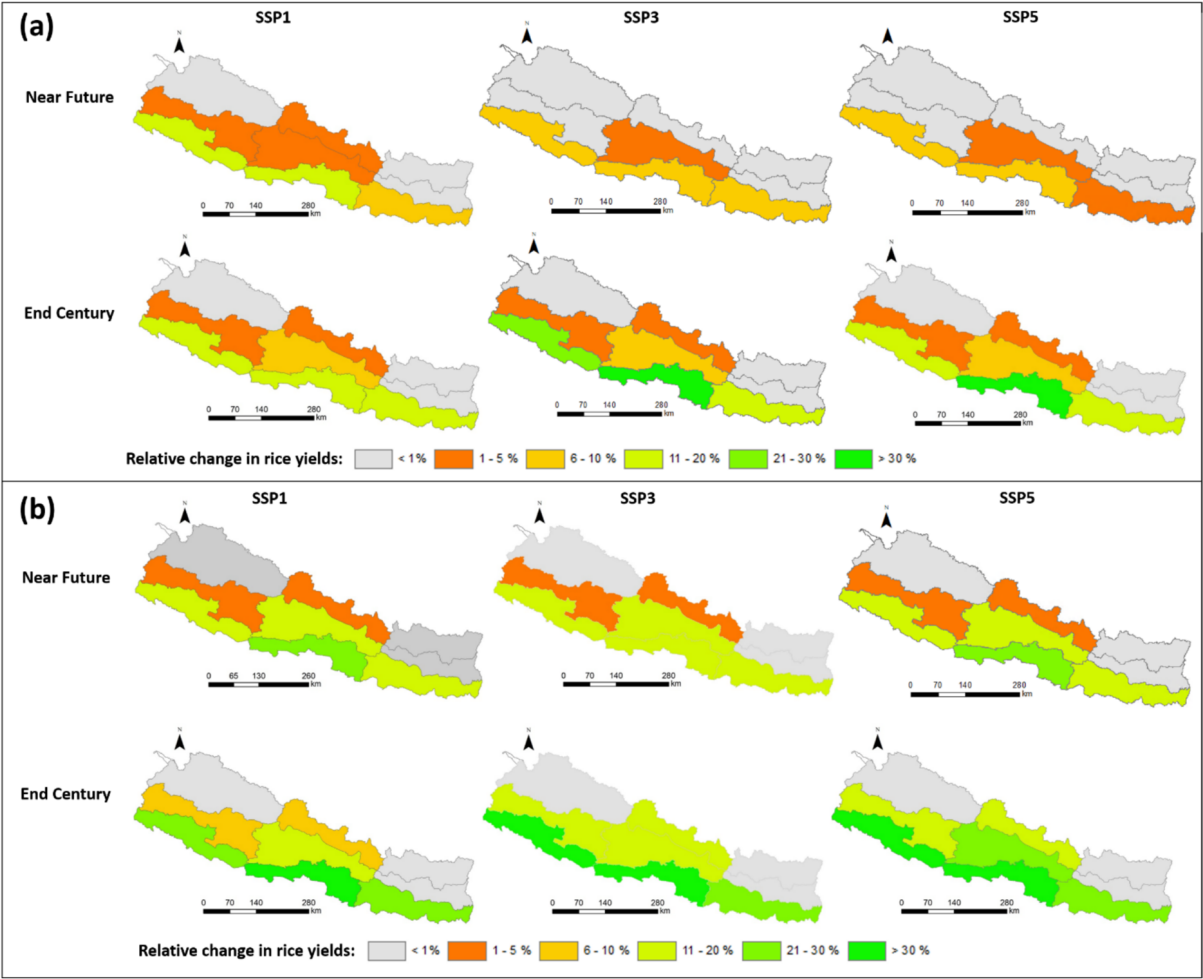
Relative changes in rice yields at ecoregion level when canal conveyance efficiency changes from 30 to 50% (**a**) and 30 to 70% (**b**) in the near and far future. SSP1, SSP3 and SSP5 represent Shared Socioeconomic Pathways with radiative forcing levels of 2.6 W/m^2^, 7.0 W/m^2^, and 8.5 W/m.^2^

Across all ecoregions, transitioning from current management practices to the adoption of irrigation expansion with a 30% conveyance efficiency (CCE) leads to a notable increase in rice yields. In the mountain region, the rice yields increase by 1.6 t/ha, in the hills by 1.6 t/ha, and in the terai region, the increase is substantially higher at 3.1 t/ha (Fig. 6a). With a further improvement of CCE, the most significant impact is observed in the terai region, where yields increase additionally by an average of 0.5 t/ha (i.e. 2–15%, Table 4) when CCE is enhanced from 30 to 50%, and by an average of 0.93 t/ha (14–77%, Table 5) when CCE is further increased from 30 to 70%. The middle terai region, in particular, exhibits the most significant benefits from increasing CCE (Fig. 7), here the average yield increases by 0.64 t/ha (4–15%) with a transition from 30 to 50% CCE, and by 1.19 t/ha (8–31%) when transitioning from 30 to 70% CCE. Conversely, there is no discernible impact on rice yield enhancement in the eastern hill region and across all mountain regions from improving CCE. This underscores that achieving a CCE of at least 50%, ideally 70%, is strongly recommended for the terai region, especially in the middle terai, for the near future. Meanwhile, for the hill and mountain regions, a 30% CCE is sufficient.

In the far future, a transition from current management to irrigation expansion with a 30% conveyance efficiency (CCE) leads to yield increases in the mountain regions of 1.2 t/ha, in the hill regions of 1.1 t/ha, and in the terai regions of 2.5 t/ha (Fig. 6b). A further enhancement of CCE yields the most pronounced impact within the terai region. In this locale, augmenting the CCE from 30 to 50% engenders an average yield augmentation of 0.8 t/ha (7–42% increment), whereas an elevation of CCE to 70% leads to a yield increment of 1.51 t/ha (14–77%). The middle terai region, in particular, witnesses the most significant yield amplifications, noted by an average increase of 1.15 t/ha (11–42%) following a CCE transition from 30 to 50%, and a further surge of 2.13 t/ha (18–77%) as CCE increases from 30 to 70%. In the middle hill region, yields increase by 0.26 t/ha (4–13%) after a 30 to 50% CCE transition, and by 0.67 t/ha (12–33%) after a 30 to 70% CCE adjustment. While yield improvements are indeed evident across other hill regions, their magnitude does not parallel the significant amplifications witnessed in the terai region (Fig. 7). The forecast for the three mountain regions shows only insubstantial yield increments of less than 5%, often less than 1% (Fig. 7). Based on the results, we conclude that a CCE of 30% is sufficient for hill and mountain regions, while the tropical terai would benefit from a CCE of 50% and more.

#### Maize

**Fig. 8:**
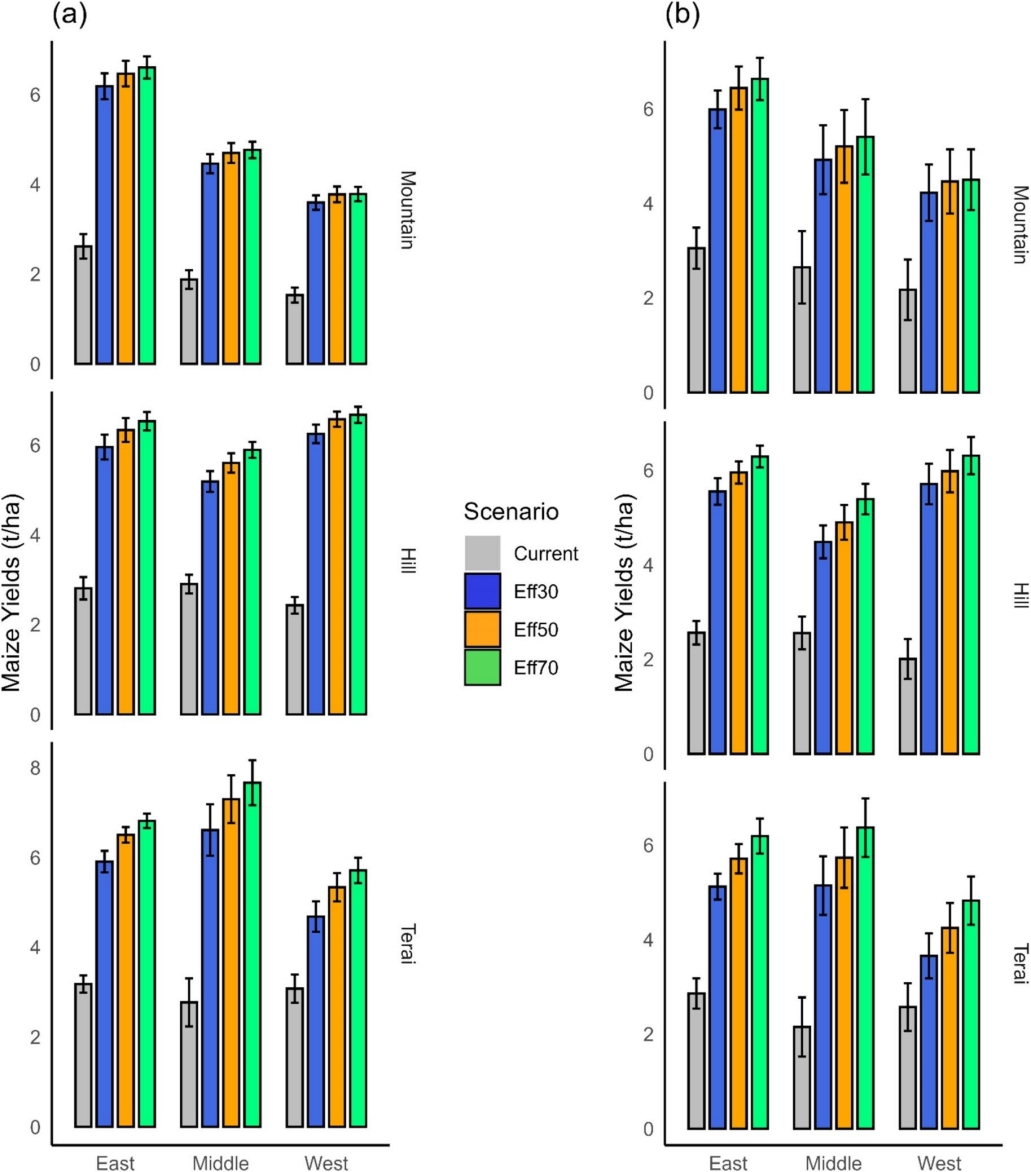
Maize yields in the near future (**a**) and far future (**b**). Eff30 = canal conveyance efficiency (CCE) of 30%, Eff50 = CCE of 50%, Eff70 = CCE of 70%. The confidence intervals denote the range over the three climate change scenarios and the three GCM models

**Fig. 9:**
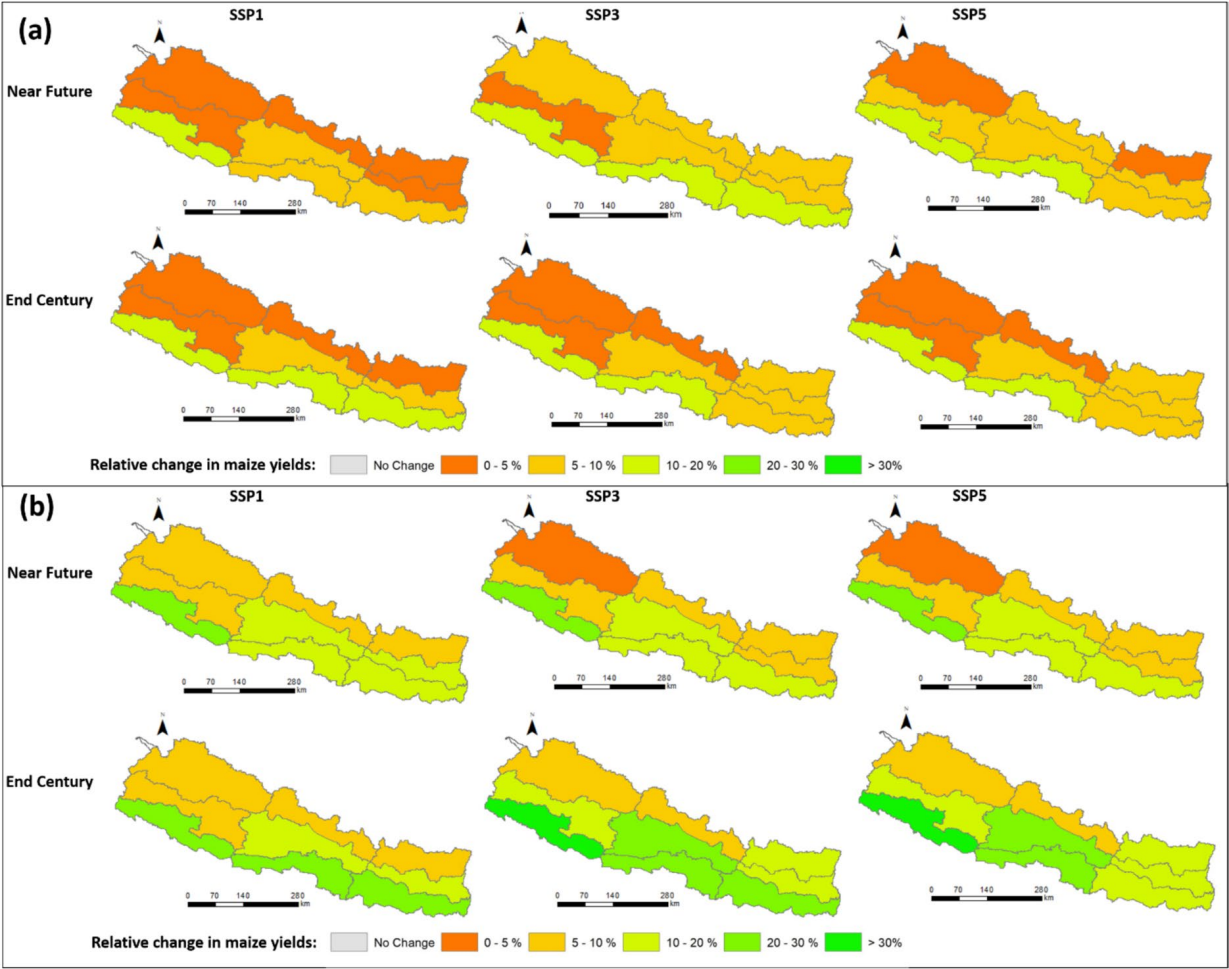
Relative changes in maize yields at ecoregion level when canal conveyance efficiency changes from 30 to 50% (**a**) and 30 to 70% (**b**) in the near and far future. SSP1, SSP3 and SSP5 represent Shared Socioeconomic Pathways with radiative forcing levels of 2.6 W/m^2^, 7.0 W/m^2^, and 8.5 W/m.^2^

Moving from existing management practices to irrigation expansion with a 30% conveyance efficiency (CCE) significantly boosts maize yields in all ecoregions. In the mountain regions, yields increase by 2.7 t/ha, in hill regions by 3.1 t/ha, and in the terai region by 2.7 t/ha respectively (Fig. 8a). The most substantial effect of enhancing CCE is seen in the terai region. Upgrading CCE from 30 to 50% across the terai results in a yield gain of on average 0.6 t/ha (7–27%, Table 6), while a further increase to 70% CCE yields an approximate rise of 1.0 t/ha (11–28%, Table 7). In the hill region, yields increase by 0.4 t/ha (3–14%) with the 30 to 50% CCE transition, and by 0.6 t/ha (5–17%) with the 30 to 70% CCE transition. For all three mountain regions, maize yield increases of less than 10% are anticipated in the near future (Fig. 9). Thus, the data indicates that for maize, expanding irrigation with a 50 to 70% conveyance efficiency is advisable in the terai regions in the near future. For other regions, a conveyance efficiency of 30% is sufficient.

Regarding maize yield by the end of the century, transitioning to irrigation with a 30% CCE from current management significantly enhances maize yields across all ecoregions (Fig. 8b). In the mountain regions, yields increase by 2.1 t/ha, in hill regions by 2.9 t/ha, and in the terai region by 2.4 t/ha. Similarly, moving from a 30 to a 50% CCE in the terai results in an average yield increase of 0.6 t/ha (7–21%, Table 6), while advancing to a 70% CCE leads to a growth of 1.2 t/ha (10–38%, Table 7). In the hill region, the yield rises by 0.4 t/ha (1–13%) with the 30 to 50% CCE transition and by 0.7 t/ha (8–25%) with the 30 to 70% CCE transition. Across all three mountain regions, yield increases of less than 10% are anticipated by the end of the century (Fig. 9). Based on these findings, expanding irrigation with a conveyance efficiency ranging from 50 to 70% is highly recommended for the terai and hill regions. For mountain regions, a conveyance efficiency of 30% is sufficient.

#### Wheat

**Fig. 10:**
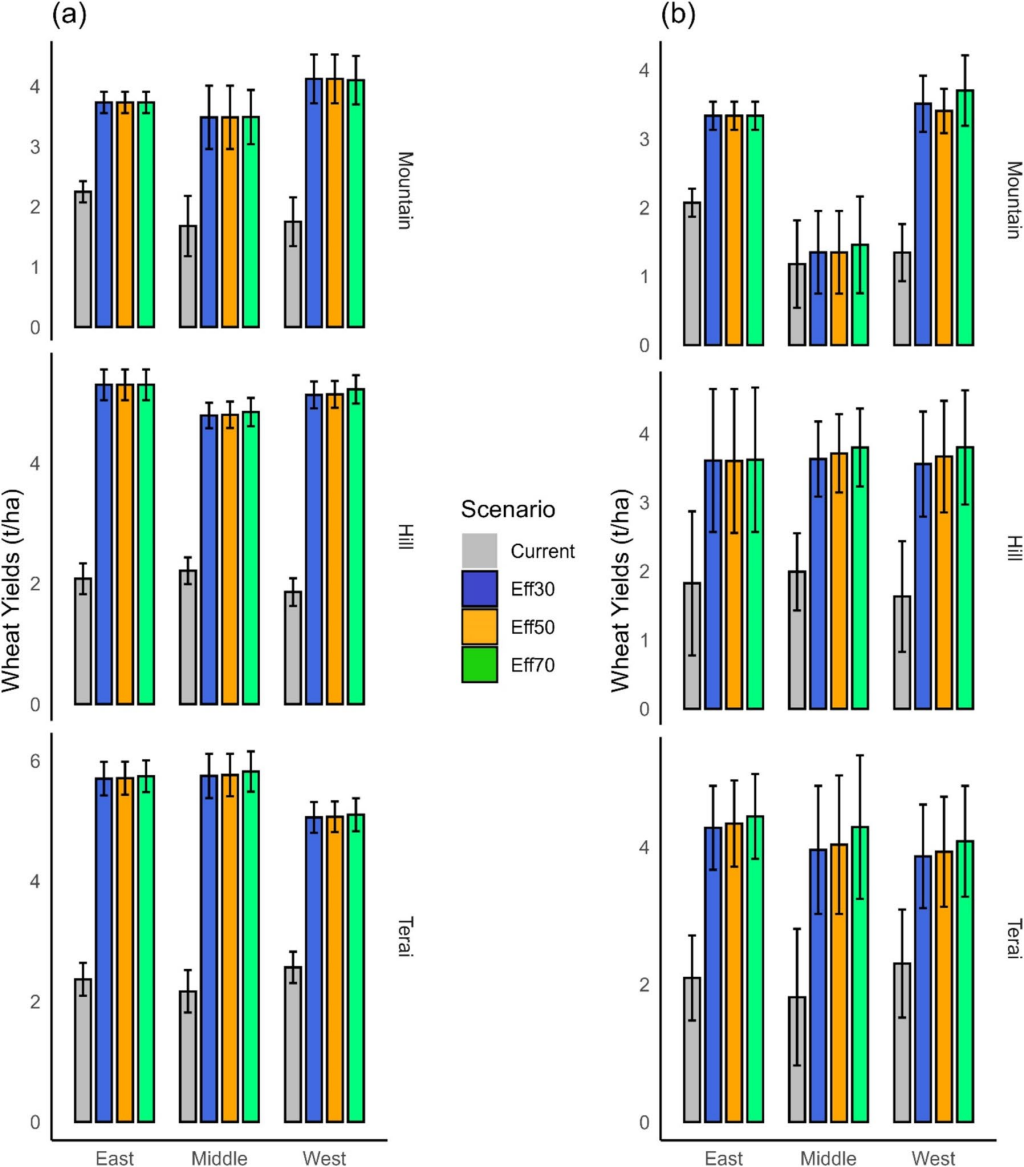
Wheat yields in the near future (**a**) and far future (**b**). Eff30 = canal conveyance efficiency (CCE) of 30%, Eff50 = CCE of 50%, Eff70 = CCE of 70%. The confidence intervals denote the range over the three climate change scenarios and the three GCM models

**Fig. 11:**
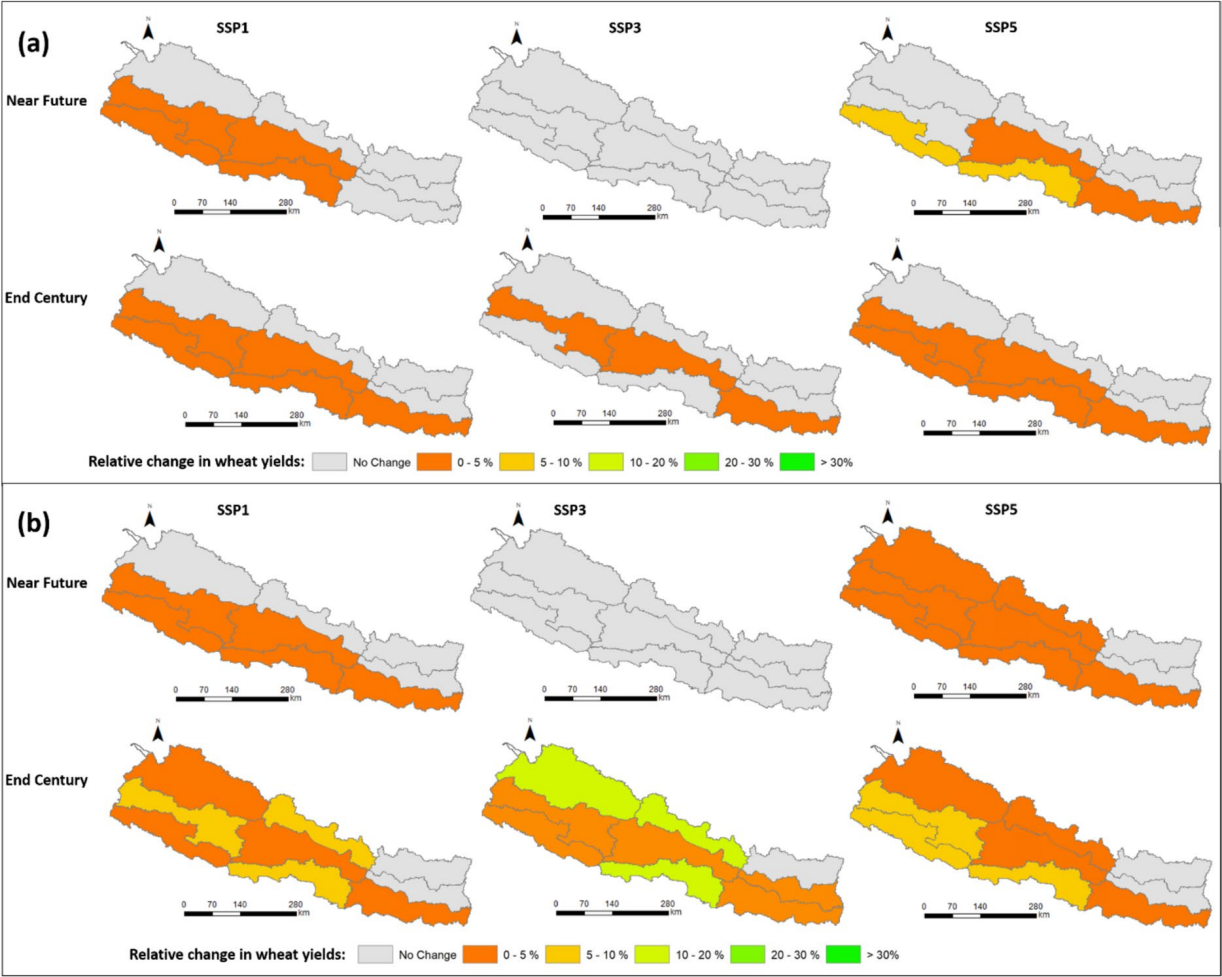
Relative changes in wheat yields at ecoregion level when canal conveyance efficiency changes from 30 to 50% (**a**) and 30 to 70% (**b**) in the near and far future. SSP1, SSP3 and SSP5 represent Shared Socioeconomic Pathways with radiative forcing levels of 2.6 W/m^2^, 7.0 W/m^2^, and 8.5 W/m.^2^

**Fig. 12:**
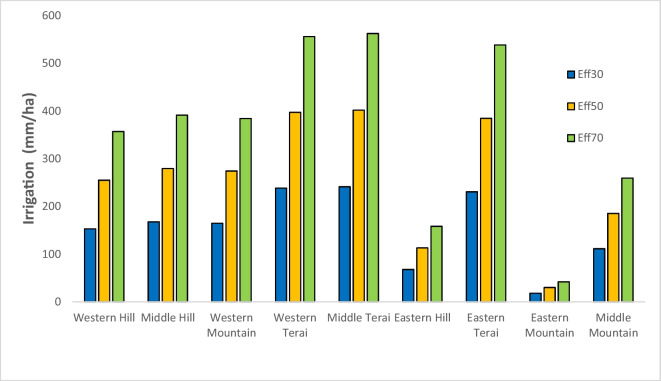
Different amounts of irrigation water conveyed to rice crops based on the canal efficiency

**Fig. 13:**
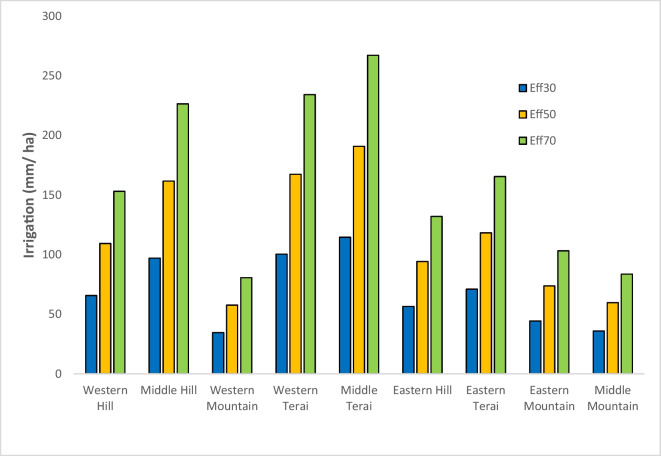
Different amounts of irrigation water conveyed to maize crops based on the canal efficiency

**Fig. 14:**
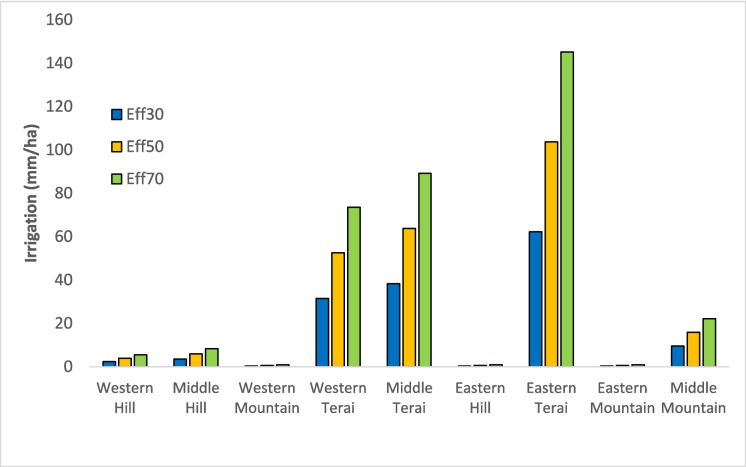
Different amounts of irrigation water conveyed to wheat crops based on the canal efficiency

**Fig. 15:**
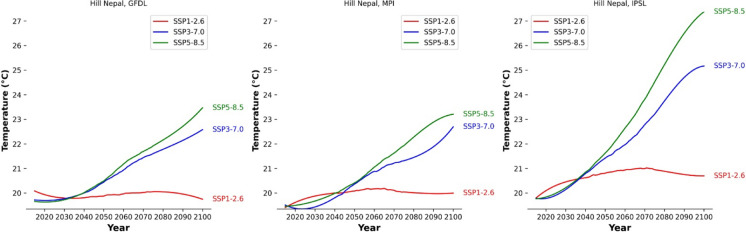
Trends of annual average temperatures in the hill ecoregion under three different climate scenarios using three different general circulation models

**Fig. 16:**
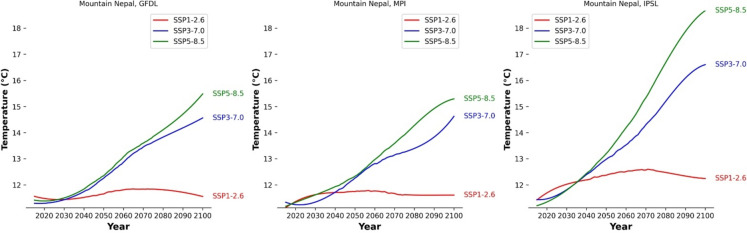
Trends of annual average temperatures in the mountain ecoregion under three different climate scenarios using three different general circulation models

**Fig. 17:**
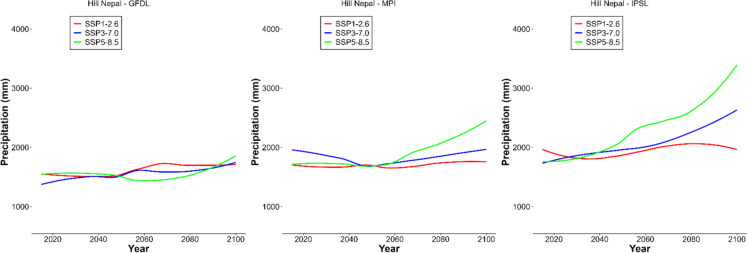
Trends of annual precipitation in the hill ecoregion under three different climate scenarios using three different general circulation models

**Fig. 18:**
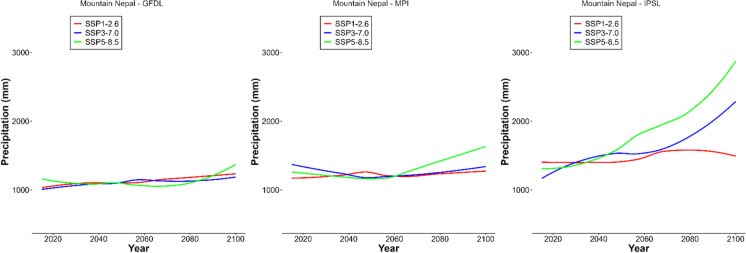
Trends of annual precipitation in the mountain ecoregion under three different climate scenarios using three different general circulation models

Switching from current practices to irrigation with a 30% conveyance efficiency (CCE) significantly increases wheat yields in all ecoregions (Fig. 10). In the mountain region, yields increase by 1.9 t/ha; in the hill region, by 3.03 t/ha; and in the terai region, by 3.13 t/ha (Fig. 9). However, in all of Nepal’s regions, improvements of less than 5% in wheat yields are expected when CCE is further enhanced to 50% in the near future (Table 8). Even an increase to 70% CCE would only benefit one region (middle mountains, with yield increases of potentially more than 10%, Table 9, Figs. 10 and 11), which shows that a conveyance efficiency of 30% is adequate for all ecoregions in the short term.

In the far future, switching from current practices to irrigation with a 30% conveyance efficiency (CCE) significantly increases wheat yield in all ecoregions. Specifically, in the western and eastern mountain regions, yields improve by 2.2 t/ha and 1.3 t/ha, respectively; in the hill regions by 1.8 t/ha; and in the terai region, by 1.96 t/ha (Fig. 10). When CCE is increased from 30 to 50%, in the terai region, yields increase only marginally further by 0.2 t/ha (0–4%, Fig. 11), and a transition from 30 to 70% CCE results in a yield increase of 0.3 t/ha (0–15%, Table 9). In the hill regions, yields increase additionally by 0.2 t/ha (0–9%) when CCE improves from 30 to 70%. In the mountain regions, increases in wheat yields range from 0 to 25% by the end of the century if CCE is increased to 70% (Fig. 11), but mean yield increases are below 5%. The findings make it clear that a conveyance efficiency of 30% is adequate for wheat production, even in the far future.

### Implications for fertilizer use

In Nepal, the current utilization of fertilizers is low across all crops (Tables 2 and 3, ‘current’), as it is often difficult for farmers to purchase fertilizers in sufficient quantities (Basukala & Rasche, 2022; Takeshima, 2019). However, to reach the growth potential offered by higher rates of irrigation, the crops demand more nutrients in the CCE scenarios. Rice may require an increase in nitrogen fertilizer of 200–250% from current rates in the near and far future, and double the rate of phosphorous fertilizer. The fertilizer requirements of maize are even higher with an increase of 300–350% of nitrogen and 500% of phosphorous fertilizer. Wheat, lastly, may require an increase of roughly 340% in the near, and 250% in the far future compared to current nitrogen fertilization rates, and an increase of 50 to 75% of phosphorous fertilizer in the near and far future, respectively. The results show that fertilization rates need to increase considerably if the yield potential is to be realized. The results also show that an increase in a canal conveyance efficiency from 30 to 70% does not markedly influence fertilization rates, and that nutrient requirements will not change noticeably between the near and far future, except for nitrogen fertilizer for wheat.

**Table 2:** Mineral nitrogen fertilizer requirements in kg/ha for three major ecoregions of Nepal under different scenarios and in different periods

		Terai			Hill			Mountain		
Period	Scenario	Rice	Wheat	Maize	Rice	Wheat	Maize	Rice	Wheat	Maize
Current	Current	60	60	60	30	27	28	21	18	20
Near future	CCE30	145	166	150	137	159	167	123	135	146
	CCE50	124	166	163	111	159	175	99	135	153
	CCE70	129	167	169	114	160	177	100	135	153
Far future	CCE30	125	139	130	130	125	158	130	99	149
	CCE50	110	140	140	107	126	166	101	99	159
	CCE70	117	144	150	110	127	170	104	102	159

**Table 3:** Mineral phosphorous fertilizer requirements in kg/ha for three major ecoregions of Nepal under different scenarios and in different periods

		Terai			Hill			Mountain		
Period	Scenario	Rice	Wheat	Maize	Rice	Wheat	Maize	Rice	Wheat	Maize
Current	Current	24	15	18	9	7	7	3	2	2
Near future	CCE30	39	18	45	18	13	57	15	5	48
	CCE50	40	15	52	18	13	59	14	7	51
	CCE70	40	15	55	18	13	61	15	7	52
Far future	CCE30	36	20	46	17	15	56	15	7	51
	CCE50	38	20	54	17	15	59	15	8	55
	CCE70	39	20	58	17	15	62	15	8	56

## Discussion

Climate change projections indicate that climatic and hydrological extremes and overall variations may increase in Nepal (Bhattarai et al., 2023). Hydrological fluxes for example may change by − 26 to + 37% (Palazzoli et al., 2015). Shifts in climate and hydrology will in turn affect crop productivity, both in positive and negative directions. Simulations project potential changes of crop production in the range of − 36 to + 18% for wheat, − 17 to + 4% for maize, and − 17 to + 12% for rice (Palazzoli et al., 2015), and − 60 to + 20% for wheat and rice (Shrestha & Shrestha, 2017). A study conducted in the middle hill region of Nepal demonstrated that the decrease in rice yield is mainly due to temperature stress (Shrestha & Shrestha, 2017). Similarly, globally, each degree Celsius of warming may reduce yields of maize by 3.4%, wheat by 2.4%, and rice by 0.3% (Deng et al., 2023). Building and maintaining sustainable irrigation practices in Nepal is thus essential for the stability of the agricultural sector. In this paper, we show that increasing the conveyance efficiency of canals can lead to substantial yield benefits, especially in the terai ecoregion, which is in line with the findings of other studies looking at water management for rice (Boonwichai et al., 2018; Houma et al., 2021; Rajwade et al., 2018; Shrestha et al., 2017; Wu et al., 2018). Likewise, the efficient supplementation of irrigation is expected to significantly boost maize yields in the coming decades (Liao et al., 2024; Quan et al., 2022; Sen, 2023).

Expanding and maintaining irrigation infrastructure is expensive, and priority regions with the highest potential return on investment should be identified. Our results show that the subtropical terai region should be a priority for investments, especially for the crops rice and maize. Irrigated area should be expanded and it should be ensured that a CCE of at least 50% is maintained. Agriculture in the hill regions would also benefit from a higher CCE, but resulting yield increases are projected to be lower, meaning that a moderate expansion of irrigated area and maintaining a CCE of 30 to 50% may be the most financially viable solution. In the mountain regions, investments should be sparse due to the considerably lower return on investments. Our findings also indicate that middle Nepal consistently demonstrates superior crop yields across all scenarios and crop types compared to both the eastern and western regions. An analysis based on CMIP6 climate models data for Nepal showed that the western and eastern parts of Nepal have high projected changes in hydro-climatic extremes and rising trends in mean annual maximum temperature and minimum temperature (Bhattarai et al., 2023), which may explain the lower yields in these regions.

For wheat, a different strategy is needed. Our results show that supplemental irrigation does not improve yields markedly. A study conducted in the Koshi basin of Nepal also showed that wheat was more vulnerable to climate change than maize or rice (Palazzoli et al., 2015), and further studies have similarly predicted no change or even a decrease in wheat yields even with supplemental irrigation (Bouras et al., 2019; Chattaraj et al., 2014; Mirgol et al., 2020; Vollset et al., 2020; Yuan et al., 2016). The authors of the studies argue that increased temperatures reduce the length of the growth phases of wheat, resulting in early maturity and lower water demand.

With higher water availability, the nutrient requirements of the plants increase as well (Gao et al., 2023; Wang et al., 2023). Appropriate fertilizer applications are needed to promote root development, enhance soil moisture and input absorption, and boost crop yield (Jiang et al., 2017). According to our simulations, especially nitrogen fertilization rates need to be increased drastically if the potential yield increases due to higher CCE are to be realized. Once local potential yields of a certain crop and variety are reached, adding more fertilizer does not increase yields further (Wang et al., 2021). It should therefore be a priority for the Nepalese government to ensure a steady and sufficient supply of affordable fertilizer and to develop efficient organic fertilization schemes (cf. Basukala & Rasche, 2022) along with investing in additional irrigation infrastructure projects on a larger scale.

We have shown that expanding irrigation and improving its efficiency can benefit farmers in Nepal. Currently, many irrigation schemes in Nepal, including those managed by farmers and agencies, jointly or privately, rely on gravity-based surface irrigation with low canal efficiency, resulting in significant water loss before reaching the farms. These schemes encounter difficulties in maintenance, water allocation, distribution, and scheduling, which impede the optimal use of water. Understanding the efficiency of canal irrigation systems is crucial for comprehensively assessing their crop yield—which is vital for future plans concerning irrigation expansion, agricultural intensification, and crop productivity increases in Nepal. This knowledge allows planners and decision-makers to take the necessary steps to address these issues. Improvements in irrigation can be achieved through proper canal linings, infrastructure upgrades, agronomic support, and enhancing irrigation services. Effective irrigation planning, operation, and management should be conducted within the context of river basins, considering hydrological boundaries as the most appropriate units for planning efforts. Additionally, implementing water measurement structures is crucial for accurately determining water allocation and diversion by irrigation systems. This facilitates an easy assessment of Canal Conveyance Efficiency (CCE) and the overall efficiency of irrigation systems. By monitoring CCE, water losses can be identified, and corrective measures can be swiftly implemented. The adoption of new irrigation technologies will also promote the sustainability of agricultural irrigation and water resource management.

As outlined in the Irrigation Master Plan 2019, strengthening coordination among water users’ associations, district irrigation development offices, and district agriculture development offices is essential for successful irrigation management. This involves providing both on-the-job and formal training programs focused on irrigation service development and management. With support from water users’ associations and district irrigation development offices, farmers can use seasonal climate forecasts and various irrigation scheduling tools to improve irrigation management.

This comprehensive analysis demonstrates the crucial role of improved irrigation efficiency in enhancing future agricultural productivity under changing climate conditions and emphasizes the strategic necessity of advancing irrigation practices to boost crop yields across Nepal. As a caveat, we want to mention some inherent constraints. The compilation of data on agricultural productivity, resource utilization, and management in Nepal was challenging due to the limited availability of aggregated and integrated data, necessitating reliance on diverse sources of varying quality. We thus adopted uniform management assumptions across different districts and diverse eco-regions, simplifying the complex decision-making processes of individual farmers and overlooking external constraints. Further, to accurately model microclimatic variations in Nepal and simulate crop yields precisely, climate and soil data of higher resolution would be preferable. In scenarios involving irrigation, we assumed that an expansion was always possible and that CCE could be improved, whereas in many regions, there are constraints that limit the expansion of irrigation systems and the improvement of conveyance efficiency. Moreover, we presume that cropping schedules remain unchanged annually across all scenarios, disregarding the dynamic nature of agricultural practices. The selection of crop type and crop production largely depends upon the local socio-economic conditions (Kaini et al., 2020a, 2020b). While farmers often practice intercropping to optimize land use, the EPIC model we used simulates the growth of only one crop at a time for each simulation unit.

## Conclusions

In this study, we assess the impact of expanded irrigation and varying canal conveyance efficiencies (CCE) on the production of key agricultural crops—rice, wheat, and maize—under shifting climatic conditions across Nepal’s diverse landscapes. Understanding the nuances of irrigation efficiency and its effect on crop yields is crucial for the effective management of irrigation water. We show that the benefits of improved irrigation efficiency vary by location, affecting crop yields differently across regions. Our findings underline the importance of not only constructing new irrigation projects but also rehabilitating existing irrigation schemes and upgrading infrastructure to enhance efficiency. Nationally, expanding irrigation and improving CCE to 30% can significantly boost rice, wheat, and maize yields by more than 3 t/ha, making it a highly recommended strategy for all regions. In the terai region, elevating CCE to 70% would further substantially increase crop yields, whereas in the hills, a CCE improvement to 50% is sufficient. These insights can inform decision-makers where to prioritize investments in irrigation infrastructure and where to maintain or increase the levels of CCE. Our findings also emphasize the interdependence of irrigation and fertilizer availability in agricultural development. While irrigation schemes provide significant benefits, their full potential is only realized when farmers have consistent access to affordable fertilizers. Given this crucial relationship, we recommend that the Nepalese government prioritize policies ensuring a reliable and cost-effective supply of fertilizers alongside irrigation initiatives. Lastly, to build a strong foundation for new agricultural schemes in Nepal, future research in the field should explore the social, cultural, economic, and agronomic factors that influence farmers’ decision-making processes. This holistic approach will help ensure that proposed schemes are not only technically sound but also culturally appropriate, socially acceptable, and financially viable.

## Data Availability

Data will be made available on request.

## Appendix

Figure 12

Figure 13

Figure 14

Figure 15

Figure 16

Figure 17

Figure 18

Table 4

**Table 4:** Aggregated rice yield changes across nine ecological regions in Nepal, showing the percentage change in yields when canal conveyance efficiency is improved from 30 to 50%, for both near future and end-of-century. SSP1_2.6, SSP3_7.0, and SSP5_8.5 represent the Shared Socioeconomic Pathways with radiative forcing levels of 2.6 W/m^2^, 7.0 W/m^2^, and 8.5 W/m^2^, respectively

	Near future				End century		
		SSP1-2.6	SSP3-7.0	SSP5-8.5	SSP1-2.6	SSP3-7.0	SSP5-8.5
Terai	East	6–10%	4–8%	2–7%	7–14%	10–12%	12–14%
	Middle	8–15%	7–12%	4–11%	11–24%	20–37%	23–42%
	West	8–14%	5–11%	4–12%	12–19%	17–26%	18–25%
Mountain	East	0%	0%	0%	0%	0%	0–1%
	Middle	0–3%	0–1%	0%	0–6%	3–6%	3–6%
	West	0%	0%	0%	0%	0%	0%
Hill	East	0%	0%	0%	0%	0%	0%
	Middle	4–6%	3–5%	2–6%	5–7%	4–11%	6–13%
	West	0–2%	0–2%	0–2%	1–4%	2–6%	3–5%

Table 5

**Table 5:** Aggregated rice yield changes across nine ecological regions in Nepal, showing the percentage change in yields when canal conveyance efficiency is improved from 30 to 70%, for both near future and end-of-century. SSP1_2.6, SSP3_7.0, and SSP5_8.5 represent the Shared Socioeconomic Pathways with radiative forcing levels of 2.6 W/m^2^, 7.0 W/m^2^, and 8.5 W/m^2^, respectively

	Near future				End century		
		SSP1-2.6	SSP3-7.0	SSP5-8.5	SSP1-2.6	SSP3-7.0	SSP5-8.5
Terai	East	12–25%	9–19%	5–15%	14–30%	23–31%	25–32%
	Middle	14–31%	12–22%	8–20%	18–43%	40–74%	44–77%
	West	11–26%	7–16%	6–20%	16–34%	27–45%	26–43%
Mountain	East	0%	0%	0%	0%	0%	0%
	Middle	2–9%	0–5%	0–2%	3–12%	8–16%	15–10%
	West	0%	0%	0%	0%	0–2%	0–1%
Hill	East	0%	0%	0%	0%	0%	0%
	Middle	10–16%	10–12%	7–13%	12–17%	12–26%	18–33%
	West	2–8%	2–6%	1–6%	5–12%	9–17%	10–16%

Table 6

**Table 6:** Aggregated maize yield changes across nine ecological regions in Nepal, showing the percentage change in yields when canal conveyance efficiency is improved from 30 to 50%, for both near future and end-of-century. SSP1_2.6, SSP3_7.0, and SSP5_8.5 represent the Shared Socioeconomic Pathways with radiative forcing levels of 2.6 W/m^2^, 7.0 W/m^2^, and 8.5 W/m^2^, respectively

		Near future			End century		
		SSP1-2.6	SSP3-7.0	SSP5-8.5	SSP1-2.6	SSP3-7.0	SSP5-8.5
Terai	East	9–10%	8– 15%	7–15%	12–14%	11–14%	7–11%
	Middle	8–10%	8–16%	7–22%	9–12%	11–13%	10–14%
	West	13–15%	11–13%	11–27%	15.5–16%	15–20%	13–21%
Mountain	East	3–4%	3–10%	2–9%	3.5–4%	5–21%	5–17%
	Middle	4–5%	4–11%	3–10%	5–6%	5–15%	0–7%
	West	3.5–4%	3–14%	2–10%	3–4%	3–16%	4–8%
Hill	East	4–6%	4–13%	3–12%	6–8%	5–15%	5–10%
	Middle	7–8%	7–8%	6–14%	9–10%	5–13%	6–13%
	West	4–5%	3–9%	3–11%	4.5–5%	2–7%	1–8%

Table 7

**Table 7:** Aggregated maize yield changes across nine ecological regions in Nepal, showing the percentage change in yields when canal conveyance efficiency is improved from 30 to 70%, for both near future and end-of-century. SSP1_2.6, SSP3_7.0, and SSP5_8.5 represent the Shared Socioeconomic Pathways with radiative forcing levels of 2.6 W/m^2^, 7.0 W/m^2^, and 8.5 W/m^2^, respectively

		Near future			End century		
		SSP1-2.6	SSP3-7.0	SSP5-8.5	SSP1-2.6	SSP3-7.0	SSP5-8.5
**Terai**	East	15–19%	14–18%	11–17%	22–27%	17–24%	10–23%
	Middle	15–21%	15–17%	13–18%	19–25%	21–26%	23–29%
	West	22–28%	18–23%	19–24%	29–31%	28–35%	30–38%
**Mountain**	East	7–9%	6–8%	5–7%	9–10%	11–12%	11–13%
	Middle	7–10%	6–7%	5–7%	8–11%	9–10%	9–13%
	West	6–7%	4–5%	4–6%	6–7%	5–7%	6–8%
**Hill**	East	9–12%	8–11%	6–10%	13–17%	12–15%	9–14%
	Middle	14–17%	13–14%	11–14%	17–20%	18–25%	19–24%
	West	7–9%	6–7%	5–8%	8–10%	8–13%	9–14%

Table 8

**Table 8:** Aggregated wheat yield changes across nine ecological regions in Nepal, showing the percentage change in yields when canal conveyance efficiency is improved from 30 to 50%, for both near future and end-of-century. SSP1_2.6, SSP3_7.0, and SSP5_8.5 represent the Shared Socioeconomic Pathways with radiative forcing levels of 2.6 W/m^2^, 7.0 W/m^2^, and 8.5 W/m^2^, respectively

		Near future			End century		
		SSP1-2.6	SSP3-7.0	SSP5-8.5	SSP1-2.6	SSP3-7.0	SSP5-8.5
**Terai**	East	0–1%	0%	0%	0–1%	0–2%	1.5–2%
	Middle	0–2%	0%	0%	2.5–3%	0–3%	3–4%
	West	0–2%	0%	0%	2.5–3%	0–2%	2–4%
**Mountain**	East	0%	0%	0%	0%	0%	0%
	Middle	0%	0%	0%	0%	0%	0%
	West	0%	0%	0%	0%	0%	0%
**Hill**	East	0%	0%	0%	0%	0%	0%
	Middle	0–2%	0%	0%	2–3%	0–3%	2.5–3%
	West	0–2%	0%	0%	2–5%	0–4%	3.5–4%

Table 9

**Table 9:** Aggregated wheat yield changes across nine ecological regions in Nepal, showing the percentage change in yields when canal conveyance efficiency is improved from 30 to 70%, for both near future and end-of-century. SSP1_2.6, SSP3_7.0, and SSP5_8.5 represent the Shared Socioeconomic Pathways with radiative forcing levels of 2.6 W/m^2^, 7.0 W/m^2^, and 8.5 W/m^2^, respectively

		Near future			End century		
		SSP1-2.6	SSP3-7.0	SSP5-8.5	SSP1-2.6	SSP3-7.0	SSP5-8.5
**Terai**	East	0–3%	0%	0–3%	1–4%	3–6%	4–5%
	Middle	0–8%	0%	0–7%	3–9%	0–15%	8–10%
	West	0–5%	0%	0–5%	3–6%	0–9%	5–9%
**Mountain**	East	0%	0%	0%	0%	0%	0%
	Middle	0–11%	0%	0–12%	0–16%	0–15%	0–3%
	West	0–6%	0%	0–5%	0–6%	0–25%	5%
**Hill**	East	0%	0%	0%	0%	0–3%	0%
	Middle	0–4%	0%	0–4%	3–4%	4–5%	5–6%
	West	0–7%	0%	0–7%	5–8%	4–7%	7–9%

Table 10

**Table 10:** Yearly inorganic and organic fertilizer use in Nepal based on data from CBS Nepal decadal agricultural census data and Takeshima et al. (Takeshima, 2019)

Fertilizer type	Terai	Hill	Mountain
Urea (kg/ha)	28	18	12
Complex (kg/ha)	3	4	2
DAP (kg/ha)	17	3	1
Organic N fertilizer (kg/ha)	22	16	13
Organic P fertilizer (kg/ha)	11	4	2
Other inorganic (kg/ha)	0	0	0

Table 11

**Table 11:** Crop schedule for the main cereal crops grown in Nepal split by ecological zone and irrigation management. *P* plant, *T* transplant, *H* harvest

Crop	Ecological zone	Irrigation	Jan	Feb	Mar	Apr	May	Jun	Jul	Aug	Sep	Oct	Nov	Dec
Rice	Hill	Rainfed					T	T			H	H		
		Irrigated			T	T			H	H				
	Terai	Rainfed						T	T		H	H	H	
		Irrigated			T	T			H	H	H			
Maize	Mountain	Rainfed			P	P				H	H	H		
	Hill	Rainfed			P	P				H	H			
	Terai	Rainfed		P	P			H	H					
Wheat	Mountain	Rainfed					H	H					P	P
	Hill	Rainfed			H	H	H					P	P	P
	Terai	Rainfed			H	H						P	P	

Table 12

**Table 12:** Classes of elevation, slope, and soil texture used for the delimitation of the homogeneous response units in this study

Elevation classes		Soil typological units		Elevation classes	
Class	Slope (%)	Class	Dominant soil	Elevation (m)	Classification (Barnekow Lillesø, 2005)
1	< 3	1	Eutric cambisols (Be)	< 300	Lower tropical
2	3–6	2	Eutric fluvisols (Je)	300–1000	Upper tropical
3	6–10	3	Calcic cambisols (Bk)	1000–2000	Subtropical
4	10–15	4	Dystric regosols (Rd)	2000–3000	Temperate
5	15–30	5	Dystric cambisols (Bd)	3000–4000	Subalpine
6	30–50	6	Humic acrisols (Ah)	4000–5000	Alpine
7	> 50	7	Humic acrisols (Ah)	> 5000	Trans Himalayan
		8	Rankers (U)		
		9	No soils (RK2)		
		10	Lithosols (I)		
